# Disentangling Environmental Effects on the Tree Species Abundance Distribution and Richness in a Subtropical Forest

**DOI:** 10.3389/fpls.2021.622043

**Published:** 2021-03-22

**Authors:** Guang Feng, Jihong Huang, Yue Xu, Junqing Li, Runguo Zang

**Affiliations:** ^1^Key Laboratory of Biodiversity Conservation of the National Forestry and Grassland Administration, Key Laboratory of Forest Ecology and Environment of the National Forestry and Grassland Administration, Research Institute of Forest Ecology, Environment and Protection, Chinese Academy of Forestry, Beijing, China; ^2^Co-Innovation Center for Sustainable Forestry in Southern China, Nanjing Forestry University, Nanjing, China; ^3^College of Forestry, Beijing Forestry University, Beijing, China

**Keywords:** diversity maintenance, evergreen-deciduous broadleaved mixed forest, forest dynamics plot, species abundance distribution, species richness, soil nutrients, topographical characteristics

## Abstract

As a transitional vegetation type between evergreen broadleaved forest and deciduous broadleaved forest, evergreen-deciduous broadleaved mixed forest is composed of diverse plant species. This distinctive forest is generally distributed in mountainous areas with complex landforms and heterogeneous microenvironments. However, little is known about the roles of environmental conditions in driving the species diversity patterns of this forest. Here, based on a 15-ha plot in central China, we aimed to understand how and to what extent topographical characteristics and soil nutrients regulate the number and relative abundance of tree species in this forest. We measured environmental factors (terrain convexity, slope, soil total nitrogen, and phosphorus concentrations) and species diversity (species abundance distribution and species richness) in 20 m × 20 m subplots. Species abundance distribution was characterized by skewness, Berger–Parker index, and the proportion of singletons. The generalized additive model was used to examine the variations in diversity patterns caused by environmental factors. The structural equation model was used to assess whether and how topographical characteristics regulate species diversity via soil nutrients. We found that soil nutrients had significant negative effects on species richness and positive effects on all metrics of species abundance distribution. Convexity had significant positive effects on species richness and negative effects on all metrics of species abundance distribution, but these effects were mostly mediated by soil nutrients. Slope had significant negative effects on skewness and the Berger–Parker index, and these effects were almost independent of soil nutrients. Soil nutrients and topographical characteristics together accounted for 9.5–17.1% of variations in diversity patterns and, respectively, accounted for 8.9–13.9% and 3.3–10.7% of the variations. We concluded that soil nutrients were more important than topographical factors in regulating species diversity. Increased soil nutrient concentration led to decreased taxonomic diversity and increased species dominance and rarity. Convexity could be a better proxy for soil nutrients than slope. Moreover, these abiotic factors played limited roles in regulating diversity patterns, and it is possible that the observed patterns are also driven by some biotic and abiotic factors not considered here.

## Introduction

Understanding the drivers of diversity patterns is an essential objective of ecological researches and several far-reaching theories, such as the species pool hypothesis (Zobel et al., 2011), neutral theory (Hubbell, 2001), and the island biogeography theory (MacArthur and Wilson, 1967). It is important to note that both the number and the relative abundance of species in communities are the fundamental attributes of species diversity (Mcgill et al., 2007; Chao et al., 2014). Almost all ecological factors or processes acting on communities may not only affect the number of species but also regulate species relative abundance (Ulrich et al., 2015). Therefore, these details are worth considering together in biodiversity studies. The number of species is referred to as species richness (SR), while the species abundance distribution (SAD) describes the relative abundance of species compared with any single index of species diversity (Mcgill et al., 2007).

SAD has long been studied by qualitative analyses based on model fitting (Mcgill et al., 2007; Matthews and Whittaker, 2014). However, even if the models are built on specific mechanisms, model fitting alone is less effective in revealing the mechanisms underlying SAD, because a given SAD pattern could result from more than one mechanism (Mcgill et al., 2007; Chisholm and Pacala, 2010; Matthews et al., 2017). Therefore, quantitative analyses have become more popular in recent years (Qiao et al., 2015a; Ulrich et al., 2015; Arellano et al., 2017; Matthews et al., 2019). Quantifying the shape of SAD makes it feasible to better detect SAD difference and to test their drivers. Two essential components underlying the shape of SAD are species dominance (or commonness) and rarity, because communities in the nature are, to varying degrees, composed of a few highly abundant species and many rare species (Magurran, 2004; Mcgill et al., 2007). Specifically, species dominance usually refers to the extent to which the highly abundant species are numerically dominant, and species rarity refers to the proportion of species that rare species account for Magurran (2004), Simons et al. (2017). Therefore, SAD evenness depends on both dimensions.

Soil nutrients and topographical characteristics have been widely confirmed to be important determinants of forest community patterns at the local scale (John et al., 2007; Baldeck et al., 2013; Punchi-Manage et al., 2014; Qiao et al., 2015b). Nitrogen and phosphorus are key limiting nutrient elements for plant growth in terrestrial ecosystems (Du et al., 2020), especially in China’s forests (Tang et al., 2018). Topographical characteristics regulate a series of factors (e.g., light availability, microclimate, and soil properties) that directly matter to plant growth and survival (Chadwick and Asner, 2016; Fortunel et al., 2018). This implies that topographical influences on plant communities may be intrinsic. In essence, environmental conditions could be the determinants of community patterns because the differences among species are ecologically significant and community assembly follows the niche-based assembly rules. For example, floristic composition changes with soil nutrient level, if plant species differ highly in their nutrient preference. Under such assembly rules, local communities are hypothesized to be shaped by abiotic filtering and biotic interaction (Keddy, 1992), and both of them are related to environmental conditions (Cadotte and Tucker, 2017). In general, abiotic (environmental) filtering tends to be intensive in harsh or stressful conditions (Kraft and Ackerly, 2010), whereas strong competition (the biotic filtering process) usually takes place in benign or less stressful environments (Bertness and Callaway, 1994). That is, diversity maintenance to some degree depends on how species persist and coexist in given environmental conditions.

Although these niche-based processes are known for limiting species diversity by selectively filtering species, the imprints of either of them on SAD do not follow a uniform paradigm. Similar to the “extinction debt” concept in the context of studying habitat destruction (Tilman et al., 1994), species suffering from those filtering processes may persist with extremely low abundances for a period of time, but eventually go extinct because the populations are too small to be viable. Thus, it is not surprising that both of those filtering processes may regulate SAD evenness by either increasing (Maire et al., 2012; Arellano et al., 2017) or decreasing the proportion of rare species (Kunte, 2008; Matthews et al., 2019). Meanwhile, both processes may also reduce SAD evenness by making a few species overabundant (Jabot, 2009; Peña-Claros et al., 2012; Qiao et al., 2015a; Simons et al., 2017). These circumstances on the whole mirror the aforementioned complexity in ecology that one-to-one relationships between patterns and processes are rarely seen (Levin, 1995).

Subtropical evergreen-deciduous broadleaved mixed forest (EDBMF) is a transitional vegetation type between evergreen broadleaved forest and deciduous broadleaved forest in China (Wu, 1980; Ge and Xie, 2017). The EDBMF is composed of diverse woody plant species, especially that in central China (Huang et al., 2016; Yao et al., 2016). Given that these forests are generally distributed in mountainous areas with complex terrain and heterogeneous microenvironments, even at a local scale, understanding the roles of environmental conditions in regulating the species diversity pattern will be ecologically meaningful. Previous studies on this forest have revealed that topographical factors such as convexity and slope and soil nutrients such as total phosphorus and nitrogen concentrations are important drivers of species compositions and distributions (Wang et al., 2014; Huang et al., 2015; Qiao et al., 2015b). However, little is known about which, how, and to what extent environmental factors regulate the number and the relative abundance of species in local communities. To fill these gaps, using data from a 15-ha plot of an old-growth EDBMF established in southwest Hubei Province, central China, we aimed to understand the effects of topographical factors and soil nutrients on local SR and SAD patterns. Specifically, we focus on addressing the following questions:

(1) Do SR and SAD change with topographical factors and soil nutrients?
(2) What is the relative importance between topographical factors and soil nutrients in determining SR and SAD?
(3) How do topographical factors affect SR and SAD via soil nutrients?

## Materials and Methods

### Study Area

**FIGURE 1:**
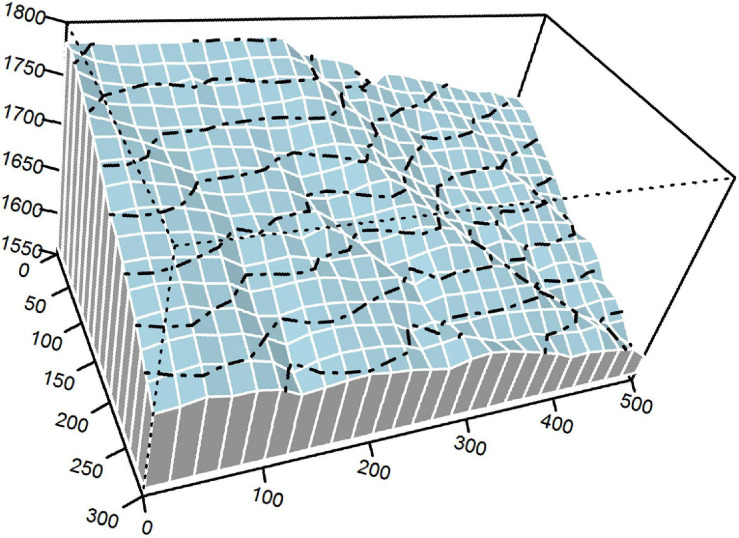
The 15-ha plot of an evergreen-deciduous broadleaved mixed forest (EDBMF). Dashed lines are contours with 20-m intervals.

Our study was conducted in a 15-ha plot of an old-growth EDBMF (300 m × 500 m; 30°4′28.50″ N, 110°12′19.30″ E; Figure 1) in Mulinzi National Nature Reserve, Southwest Hubei Province, Central China. The research area has a humid monsoon climate with a mean annual relative humidity of 82%. Mean annual precipitation is between 1,700 and 1,900 mm, and most precipitation occurs from April to September. Mean annual temperature and annual effective accumulated temperature (≥10°C) are approximately 15.5°C and 4925.4°C, respectively. Annual sunshine duration ranges from 1,253 to 1,342 h, and the frost-free period lasts for 270–279 days.

### Measurement of Tree Species Diversity

The 15-ha plot was established and divided into 375 subplots (20 m × 20 m) using a real-time kinematic method. In this plot, woody plants with diameter at breast height ≥ 1 cm were identified, tagged, and mapped in 2014. This survey included 84,171 individuals from 227 species in total. Within subplots, SR was calculated as the number of species, and SAD was characterized by skewness, Berger–Parker index (*d*), and the proportion of singletons (PS). Specifically, *d* is a dominance index (Magurran, 2004):

d=Nmax/N

where N_max_ is the number of individuals for the most abundant species and N is the total number of individuals.

PS is a rarity index (Magurran, 2004):

PS=Ssingleton/S⁢total

where S_singleton_ is the number of singletons (i.e., species represented by just one individual) and S_total_ is the total number of species.

Skewness is the well-known third moment of a probability distribution. Skewness calculated from log-transformed species abundance data depicts SAD symmetry (Magurran, 2004). Negative skewness (strong species rarity) commonly reflects that there is an excess of less-abundant species, compared with a lognormal SAD (Ulrich et al., 2015). Positive skewness (strong species dominance) reflects strong monodominance and/or an excess of abundant species compared with a lognormal SAD.

### Measurement of Environmental Factors

The following environmental factors were measured in each subplot: soil total phosphorus (TP) and total nitrogen concentrations (TN), terrain convexity, and slope (steepness). Because elevation and convexity are highly correlated, we did not consider elevation in analyses. Convexity was measured as the average elevation of the focal site minus that of its surrounding sites. Slope was calculated as the mean deviation angle from the four planes to horizontal (created in sequence by taking elevation data at three corners of each subplot). In each subplot, after removing the visible litter and humus layers, a mixed soil sample was collected at 5–15 cm depth (soils at 0–5 cm depth mixed with humus were not sampled) at three locations, including one at the center of each subplot and two along the diagonal. After the soil samples were air-dried, TP and TN were determined by the HClO_4_–H_2_SO_4_ digestion method and the semi-micro Kjeldahl method (Bao, 2000), respectively.

### Statistical Analyses

**FIGURE 2:**
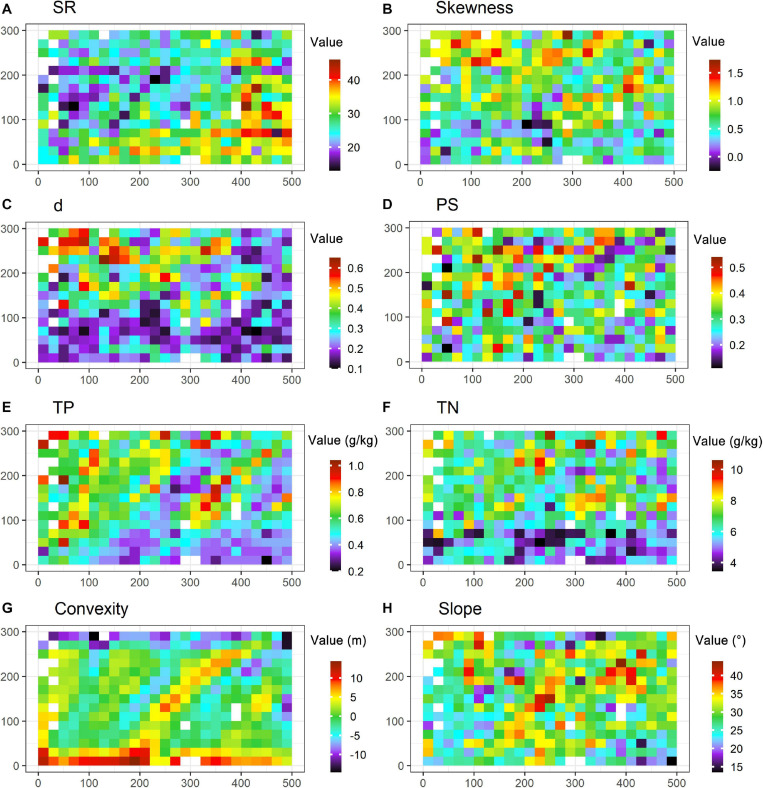
Species diversity and environmental conditions in the 15-ha plot. Subgraphs are the maps of SR (species richness, **A**), skewness **(B)**, d (the Berger-Parker index, **C**), PS (proportion of singletons, **D**), TP (soil total phosphorus, **E**), TN (soil total nitrogen, **F**), convexity **(G)** and slope **(H)** at the 15-ha plot with the resolution at 20 m × 20 m. Blanks refer to the subplots with outliers that are filtered out in analyses.

Because modeling and parameter estimation may be biased by outliers, 14 subplots with outliers were filtered out according to the three-sigma rule. The remainders (i.e., 361 subplots; Figure 2) were considered in the following analyses. Data can be viewed in Supplementary Data S1.

We first ran a principal component analysis (PCA) on environmental variables to determine the major environmental gradients across subplots and to illustrate the correlations among environmental variables, which were further quantified by Pearson correlation analysis (see Supplementary Figure S1). Similarly, pairwise correlations among diversity patterns (SR, skewness, *d*, and PS) were also evaluated by Pearson correlation analysis. The generalized additive model (GAM) was used to examine variations in diversity patterns along environmental gradients. Based on the GAM, variance partitioning was performed to quantify the variations explained by topographical factors and soil nutrients. A structural equation model (SEM) was used to disentangle whether and how topographical factors acted on diversity patterns by regulating soil nutrients. In SEM, environmental effects were measured by standardized effect size (SES). In the modeling, soil nutrients were considered as a latent variable characterized by TP and TN; soil nutrients were hypothesized to affect SAD and SR patterns directly; topographical impacts on those patterns are hypothesized to be partly mediated by soil nutrients. The overall fitting of SEM was evaluated by *p*-value (in chi-square test), goodness-of-fit index (GFI), comparative fit index (CFI), and standardized root-mean-square residual (SRMR). As a rule of thumb, the model is acceptable if *p* > 0.05 (i.e., differences between observations and fitted values are non-significant), CFI > 0.95, GFI > 0.95, and SRMR < 0.08 (Grace et al., 2016). We also ran a redundancy analysis on the highly abundant species at the plot level to examine their distributions along environmental gradients (see Supplementary Figure S2).

The above statistical analyses were carried out by using the “mgcv,” “lavaan,” “vegan,” “moments,” “stats,” and “Hmisc” packages in R software version 3.5.0 (R Core Team, 2013).

## Results

**FIGURE 3:**
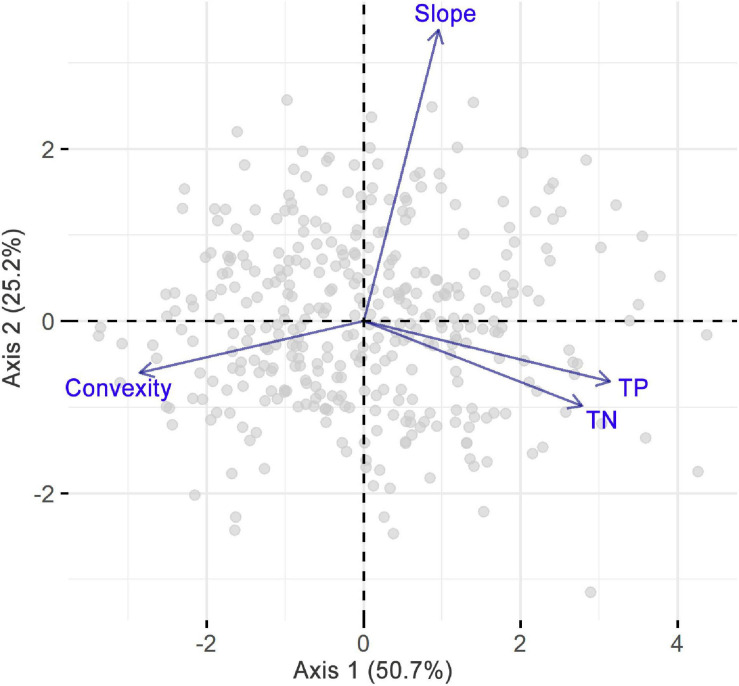
Principal component analysis on slope, convexity, soil total nitrogen (TN), and phosphorus (TP) concentrations.

**FIGURE 4:**
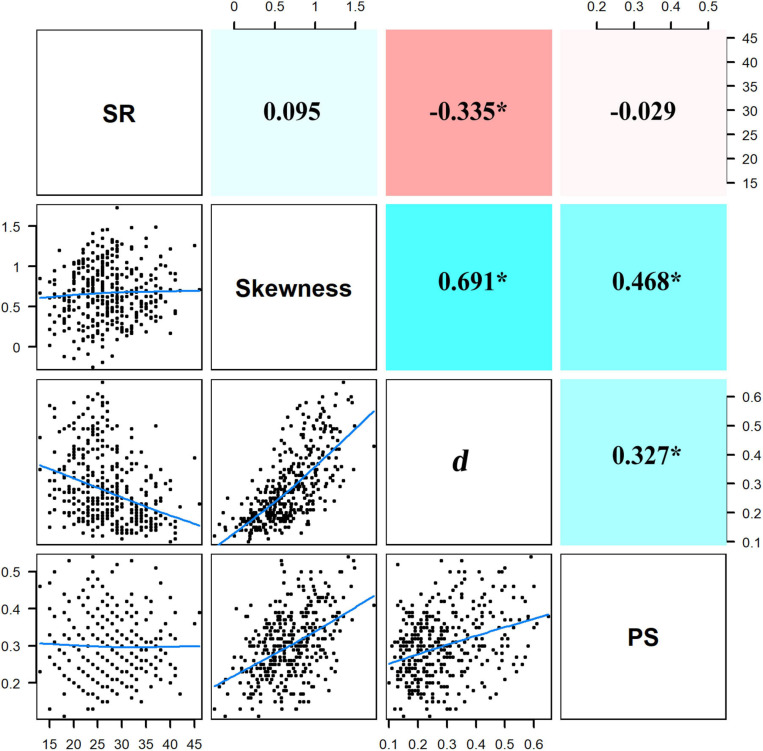
Pairwise relationships among species richness (SR), skewness, Berger–Parker index (*d*), and proportion of singletons (PS). Blue lines in the bottom left panels are the smooth curves about the pairwise relationships. Values in the upper right panels are the coefficients of correlation with significant values asterisked (*p* < 0.05); positive and negative coefficients have their background panels colored with cyan and red, respectively, larger absolute values of coefficients are in darker colors.

The first two PCA axes accounted for 75.9% of the environmental variations (Figure 3) and represented the major environmental gradients in our study area. The first axis was positively correlated with TP and TN but negatively correlated with convexity, while the second axis was mainly positively correlated with slope. Correlations among environmental factors were further explicitly examined by Pearson correlation analysis (Supplementary Figure S1), which revealed a significant positive correlation between TP and TN and a significant negative correlation between convexity and each of the other environmental variables. In addition, correlations among skewness, *d*, and PS were significantly positive, while the correlation between SR and *d* was significantly negative (Figure 4). In particular, *d* had a stronger correlation with skewness than PS, indicating that SAD symmetry is more dependent on species dominance.

**FIGURE 5:**
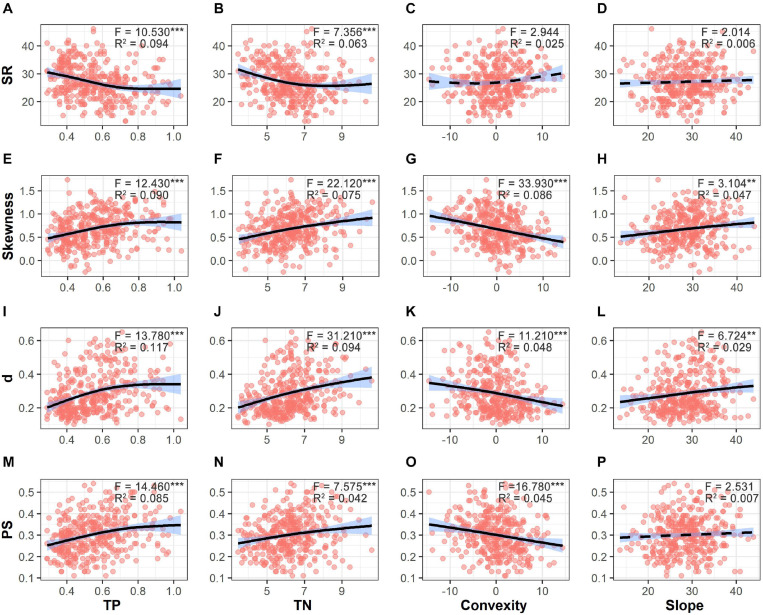
Variations in species richness (SR, **A–D**), skewness **(E–H)**, d (the Berger–Parker index, **I–L**), and PS (proportion of singletons, **M–P**) along the gradients of soil total phosphorus (TP), soil total nitrogen (TN), convexity and slope. Fitted lines are shown in black with 95% interval colored in blue. Solid lines refer to significant relationships (*p* < 0.05), while dashed lines refer to non-significant relationships. Significant level: ***p < 0.001; **p < 0.01.

**FIGURE 6:**
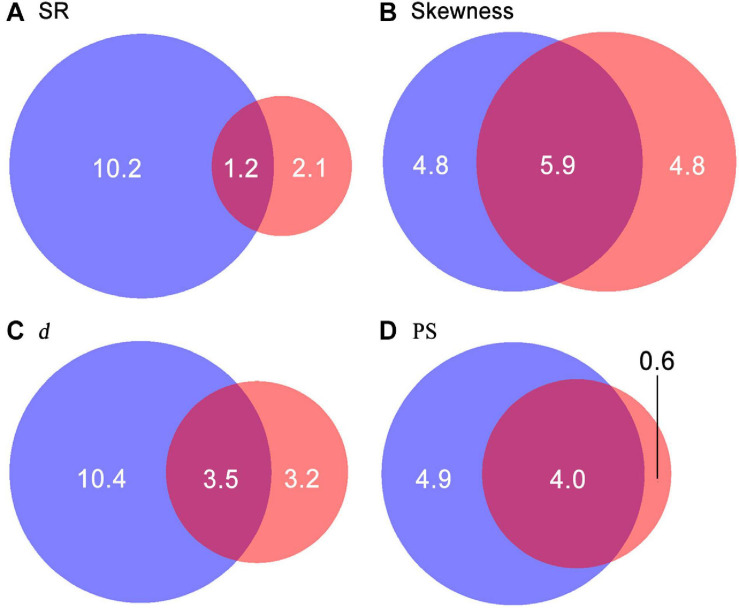
Variation partitioning for SR (species richness, **A**), skewness **(B)**, d (the Berger–Parker index, **C**), and PS (proportion of singletons, **D**). Sums of values within the blue and red circles are the percentage of variations in diversity patterns explained by soil nutrients and topographical factors, respectively. Values in the overlapping areas between the two circles are the proportions of variations jointly explained by soil nutrients and topographical factors.

SR significantly decreased along gradients of TP and TN (Figure 5). Values of SAD metrics significantly increased along gradients of TP and TN but decreased with increasing convexity. Moreover, values of skewness and *d* also showed significant increases with increasing slope degree. In total, 9.5–17.1% of variations in diversity patterns were explained by environmental factors. Only 1.2–5.9% of variations in diversity patterns were explained by both soil nutrients and topographical factors (Figure 6). Soil nutrients and topographical factors displayed the same explanatory power for skewness by accounting for 10.7% of variations; soil nutrients displayed stronger explanatory power for SR, *d*, and PS by accounting for more of their variations.

**FIGURE 7:**
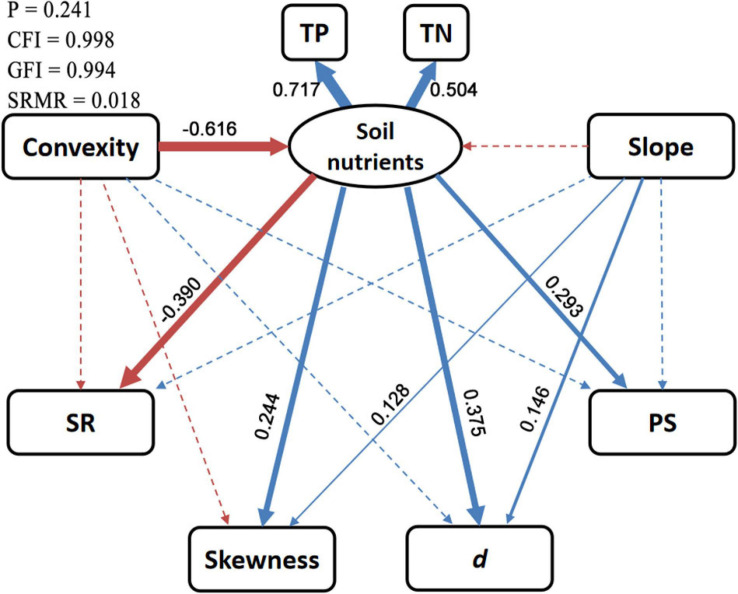
Diagrammatic sketch of structural equation model for environmental effects on diversity patterns. Solid and dashed arrows represent significant (*p* < 0.05) and non-significant direct effects, respectively. Blue and red arrows represent positive and negative effects, respectively. Standardized effect sizes (SESs) for the significant direct effects are shown beside arrows with widths proportional to the absolute values of SESs. SR, species richness; *d*, Berger–Parker index; PS, proportion of singletons; TP, soil total phosphorus; TN, soil total nitrogen. More detailed results are shown in Supplementary Table S1.

The SEM provided reliable fits to environmental effects on diversity patterns (*p* > 0.05, CFI > 0.95, GFI > 0.95, SRMR < 0.08; Figure 7 and Supplementary Table S1). Soil nutrients had significantly negative effects on SR (SES = −0.39) and positive effects on SAD metrics (SES = 0.24–0.38). The total effects of slope on skewness (SES = 0.11) and *d* (SES = 0.12) are significant, and such effects were almost independent of soil nutrients. The total effects of convexity on SR (SES = 0.12) and all SAD metrics (SES = −0.27 to −0.19) were also significant, but these effects were mostly mediated by soil nutrients. In addition, convexity also negatively effected SR (SES = −0.12) and skewness (SES = −0.12), independent of soil nutrients, but these effects were only significant at the 10% level.

## Discussion

At local scales, topographical factors and soil nutrients have been widely recognized as significant determinants of forest community features (Baldeck et al., 2013; Jucker et al., 2018; Zuleta et al., 2018; Sellan et al., 2019; Chun et al., 2020). In EDBMF, these factors turn out to be the important drivers for species distribution and composition (Wang et al., 2014; Huang et al., 2015; Qiao et al., 2015b; Wang et al., 2017). However, it remains poorly understood how tree species dominance, rarity, and richness in this forest vary in response to topographical factors and soil nutrients. Such information was investigated in the present study using data from a 15-ha plot to inform scientific understanding of the role of environmental conditions in the local species diversity maintenance in EDBMF.

Our results showed that SAD attributes varied with either topographical factors or soil nutrients, whereas SR only exhibited significant variations along soil nutrient gradients. The resulting weak SR–topography relationship is consistent with the recent finding in a tropical forest (Rodrigues et al., 2020). We further discovered that soil nutrients accounted for more variations in species dominance, rarity, and richness than topographical factors, reflecting a relatively greater importance of soil nutrients in governing local diversity patterns. Specifically, increasing soil nutrient levels led to decreases in taxonomic diversity and increases in species dominance and rarity. Similar outcomes evidenced in several ecosystems (Midolo et al., 2018), especially in forest (Peña-Claros et al., 2012) and grassland (Simons et al., 2017), have been attributed to plant competitions.

Plant competition has long been assumed to depend on soil nutrients, which are the limiting factors for plant growth and community productivity. A resource-implicit hypothesis suggests that total competition intensity increases with productivity (Grime, 1973). A resource-explicit hypothesis states that plant competitions would shift from below- to above-ground parts as productivity increases, and then species diversity becomes more limited by the intensive above-ground competitions for light (Newman, 1973; Tilman, 1988). In essence, along with individual growth, plants are more engaged in competing for nutrients because of the ever-growing requirements, and/or for light as neighborhood shading becomes more prevalent. These circumstances underlie the intense competitions in the productive communities. Many studies on forests have revealed strong light limitation in nutrient-rich sites caused by canopy shading (Coomes and Grubb, 2000; Palmiotto et al., 2004; Coomes et al., 2009). This could be the reason why the resource-explicit hypothesis has been widely advocated (DeMalach et al., 2016, 2017; Harpole et al., 2016).

Intensive interspecific competitions could reduce taxonomic diversity and SAD evenness (Grime, 1973; Arellano et al., 2017). Under such competitions, some of the less-competitive species are excluded within a short time, while the others have their population sizes limited to low levels and then face a high risk of going extinct locally. Only a few competitive species are able to be dominant. Interestingly, there is a long-standing debate on what underlies the competitive ability of plants (Bengtsson et al., 1994). *Eurya alata*, known for its shade tolerance, conservative resource use, and slow growth (Tang et al., 2016), was the only species that highly predominated in the nutrient-rich sites of our study area (Supplementary Figure S2). This finding supports the standpoint that slow-growing species with conservative resource use and strong tolerance to low resource levels can be the superior competitors (Tilman, 1982; Dybzinski and Tilman, 2007; Atwater et al., 2020). A demographic trade-off among trees denotes that fast-growing species are generally intolerant to low resource levels and more vulnerable to competition (Shipley and Keddy, 1988; Kobe and Vriesendorp, 2011; Rüger et al., 2020). Hence, slow-growing, tolerant tree species are more likely to predominate in forest communities, especially over the long run.

Topographical effects on forest communities are mediated by several factors related to, e.g., thermal, hydrologic, and edaphic conditions (Moser et al., 2009; Punchi-Manage et al., 2014; Fortunel et al., 2018). In our study, convexity showed significant effects on SAD and SR via soil nutrients, but slope did not; specifically, decreased convexity enriched soil nutrients and then reduced SAD evenness and SR, while soil nutrients hardly changed with slope. Hydrologic leaching and soil erosion may be the processes making low-lying conditions more nutrient-rich (Chadwick and Asner, 2016; Jucker et al., 2018). In addition, topographical factors were also influential in a way that was independent of soil nutrients. Increasing steepness was found to result in strong species dominance. This may be due to the large surface area of steep sites (Wang et al., 2014) or to the strong environmental heterogeneity. Slope *per se* is a measure of topographical heterogeneity (Stein et al., 2014). Heterogeneous conditions consist of various micro-habitats that favor prevalence of generalists in resource use (Batáry et al., 2007). However, increased slope only led to a slight SR increase, hardly supporting for the hypothesis that diverse species can coexist in environmentally heterogeneous conditions (Stein et al., 2014). Moreover, convexity exhibited non-significant but noticeable negative effects on species dominance and richness. This probably relates to the typical increase in soil thickness with decreasing convexity (Fortunel et al., 2018), because thick soil layers may promote the persistence and coexistence of species with varying root depths (Levine and HilleRisLambers, 2009).

Topographical factors should be used as proxy variables (for soil properties as well as for other factors) with caution, as suggested by our findings. We found non-significant SR variations along the convexity gradient, but convexity showed noticeable positive and negative effects on SR via different pathways. This indicates that investigating diversity variations along topographical gradients is inefficient for revealing topographical effects on diversity patterns. Therefore, it is essential to consider the directly effectual environmental factors for studying environmental impacts on forest communities, or else it may result in misunderstandings. Zuleta et al. (2018) found that soil properties were less heterogeneous across a 25-ha tropical forest plot and varied weakly along topographical gradients; the authors attributed this to the homogeneity of parent material, which should be common at a local scale. In such cases, the effect of soil factors will be overestimated if topographical factors are used as proxy variables for that. In a 100-ha tropical forest plot, Hall et al. (2004) observed that none of the focal species showed any preferences for topographical conditions, but most of them were significantly associated with soil properties. In our study, convexity was a surrogate for soil nutrients, but slope was not, suggesting the need to evaluate the applicability of individual topographical factors for being the proxy variable.

Environmental factors considered in this study accounted for a fraction of variation in diversity patterns. This may be ascribed to the spatial scale at which the environmental effects and species diversity were examined. From the perspective of grain size, forest community features are of strong variability across small quadrats, and this may obscure the variations along environmental gradients (Spake et al., 2020). In consideration of spatial extent, it is plausible that forest community features do not change remarkably along the relatively short environmental gradients within a local scale (<1 km^2^). As such, it is not surprising that environmental factors play limited roles in shaping small-scale community features. At the 20 m × 20 m local scale, for example, the impacts of resource quantity (i.e., a group of various resource factors) on SR patterns in two species-rich forests were studied by Zhang et al. (2020), who found that resource quantity only accounted for 0.8% of SR variation in a 50-ha tropical forest plot in Barro Colorado Island and for 4.9% of that in a 24-ha subtropical forest plot in Gutianshan.

It is also noteworthy that we only considered the roles of a few abiotic factors, and some biotic and other abiotic factors (e.g., microclimate and light conditions) that are not considered here may act on diversity patterns as well. In particular, the diversity maintenance mechanisms could be various and based on different ecological processes, but not all of them underlie how environmental conditions affect diversity patterns. At small scales, population dynamics and species turnovers are assumed to be susceptible to ecological drifts (Shipley et al., 2012), immigration events (Volkov et al., 2007), and some processes taking place among neighbors. This denotes the potential importance of neutral processes and negative density dependence on diversity maintenance. To better decipher the diversity maintenance mechanisms of EDBMF, exploring the influences of the other potential factors and/or processes on diversity patterns would be a significant step forward.

## Conclusion

In our study area, SR and SAD varied along gradients of topography and soil nutrients, while soil nutrients exhibited stronger influences than topographical factors on these patterns. Forest communities tended to show low taxonomic diversity and strong species dominance and rarity in nutrient-rich conditions. Convexity mainly acted on diversity SR and SAD patterns via soil nutrients, while slope had positive effects, barely related to the effects of soil nutrients, on species dominance. Topographical factors and soil nutrients played significant but limited roles in local diversity maintenance. Presumably, the limited roles of environmental factors can be attributed to the fact that environmental effects were studied at small scales, but this also indicates the potential importance of the other drivers for the observed diversity patterns.

## Data Availability Statement

The original contributions presented in the study are included in the article/Supplementary Material, further inquiries can be directed to the corresponding author/s.

## Author Contributions

JL, RZ, and JH designed the research project and provided theoretical guidance. GF collected the data. GF analyzed the data and wrote the first draft with the help of YX. All authors approved the final submission.

## Conflict of Interest

The authors declare that the research was conducted in the absence of any commercial or financial relationships that could be construed as a potential conflict of interest.
