# Supplementary figures and images for: Disentangling Environmental Effects on the Tree Species Abundance Distribution and Richness in a Subtropical Forest
Source: Front Plant Sci. 2021 Mar 22;12:622043. doi: 10.3389/fpls.2021.622043 (PMC8020568; doi:10.3389/fpls.2021.622043)

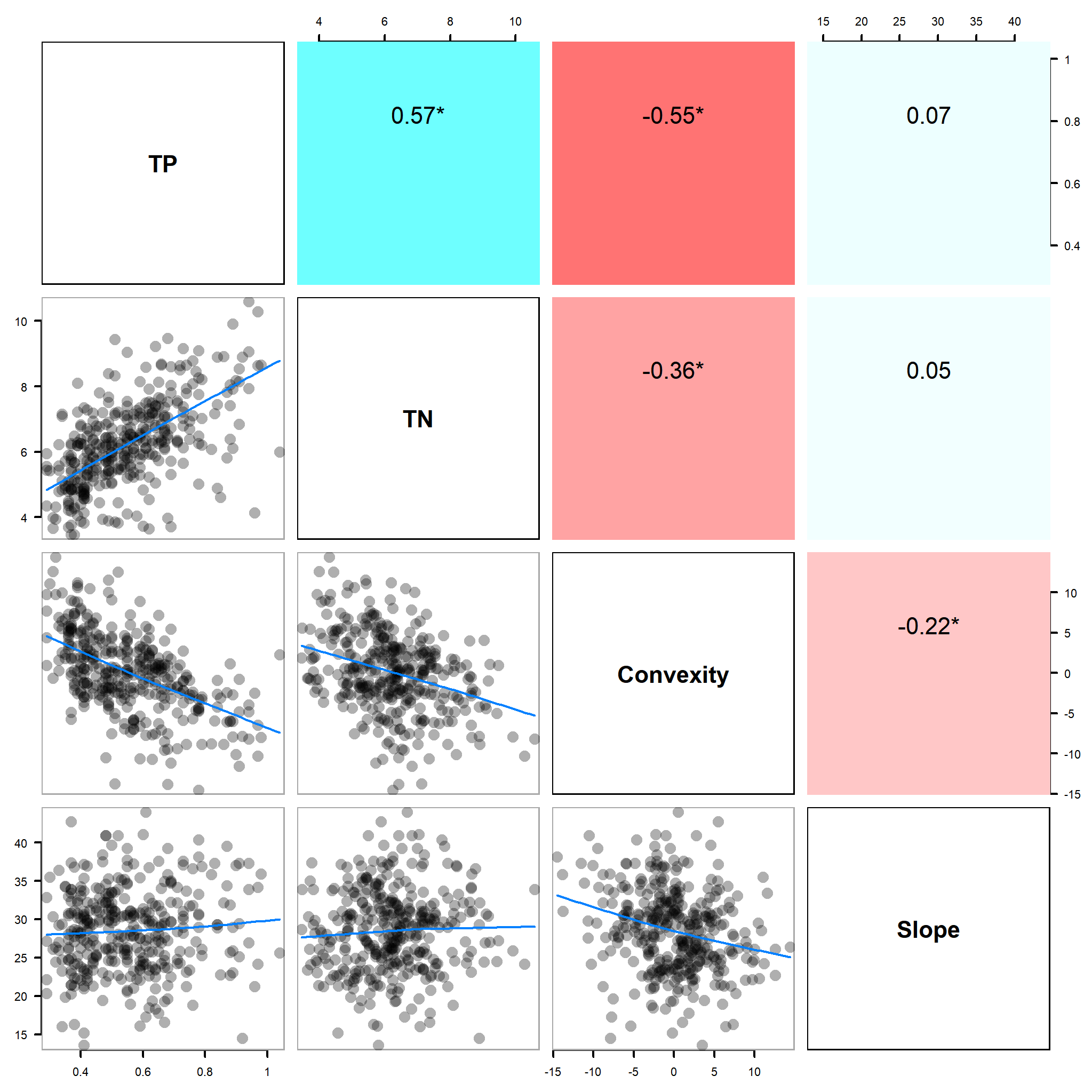

Supplement: Supplementary file 2 [file Image_1.TIFF]

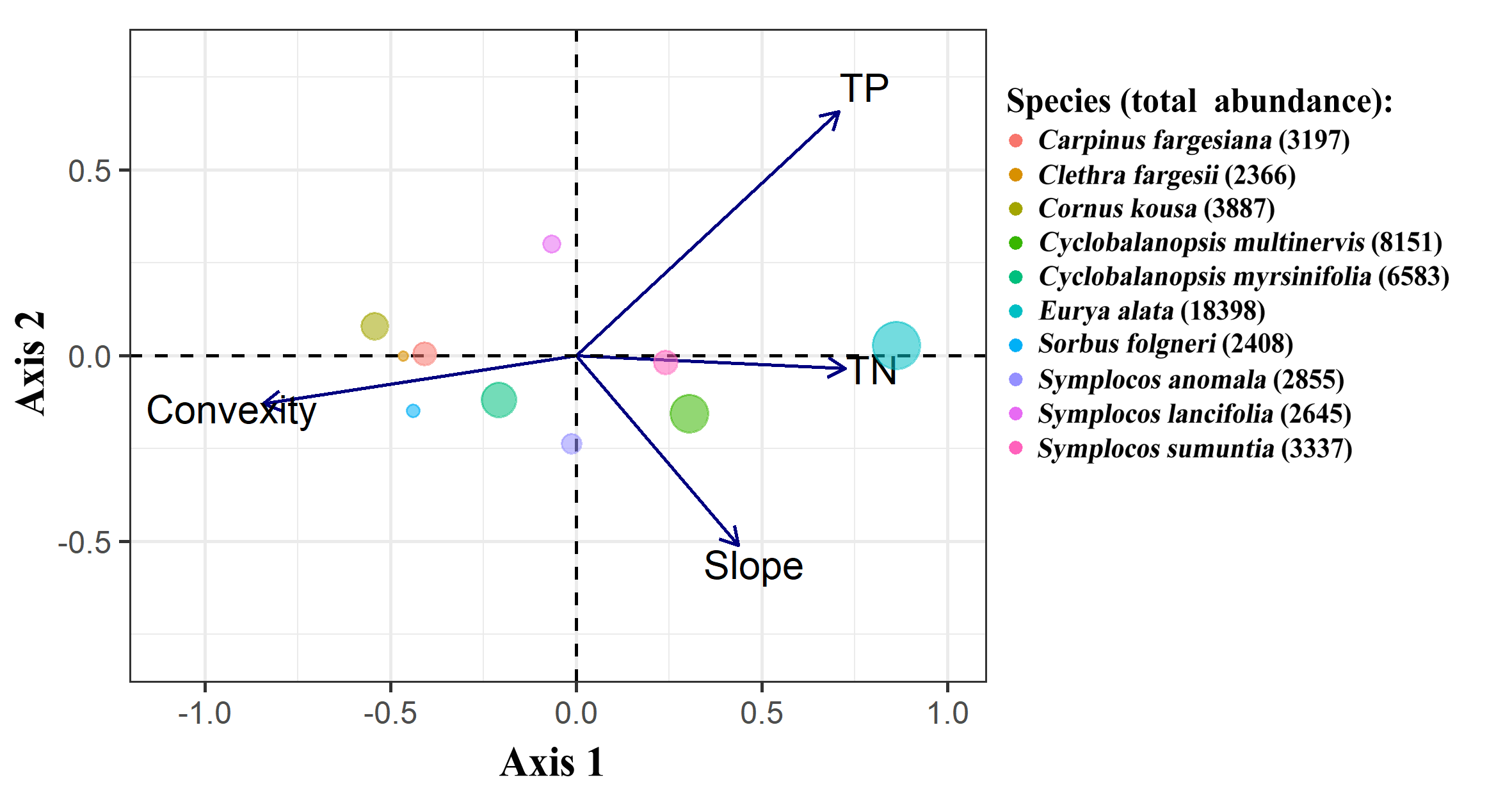

Supplement: Supplementary file 3 [file Image_2.TIFF]
